# Prevalence and association of compliance with the Canadian 24-hour movement guidelines with sociodemographic aspects in Brazilian adults: a cross-sectional epidemiological study

**DOI:** 10.1186/s12889-024-17720-w

**Published:** 2024-01-22

**Authors:** Leandro Dragueta Delfino, William Rodrigues Tebar, Diego Giulliano Destro Christofaro

**Affiliations:** 1 Graduate Program in Movement Sciences, Physical Education Department, Faculty of Science and Technology, São Paulo State University - UNESP Presidente Prudente, 19060-900 São Paulo, SP Brazil; 2 Center of Clinical and Epidemiological Research, University Hospital, University of São Paulo - USP, 05403-000 São Paulo, SP Brazil

**Keywords:** Guideline adherence, Physical activity, Sedentary behavior, Sleep duration, Socioeconomic factors

## Abstract

**Background:**

The physical inactivity is a global health concern, so that recommendations on sufficient physical activity levels are elaborated worldwide, such as in Brazil. However, the Canadian 24-Hour Movement Guidelines were first in the world to consider time-specific recommendations for physical activity, sedentary behavior and sleep, which is still not developed for Latin-American population. The present study aimed to verify the adherence to Canadian 24-hour guidelines in a Brazilian inner city and to analyze its association with sociodemographic aspects.

**Methods:**

A cross-sectional epidemiological study, with a sample composed by 250 adults (140 women), with a median age of 41.0 years. Objective measures of moderate-to-vigorous physical activity (MVPA) and sedentary behavior were collected by accelerometry. Sleep duration and sociodemographic aspects (ethnicity, gender, age, educational attainment and socioeconomic level) were obtained through a face-to-face questionnaire. Canadian 24-hour guidelines considered ≥ 150 min/week of MVPA, <8 h/day of sedentary behavior and daily sleep time between 7 and 9 h, being analyzed separately and in combination. Poisson regression with robust variance estimator was used to analyze the prevalence ratio (PR) of meeting the 24-hour guidelines according to the categories of sociodemographic variables, being performed by the software IBM SPSS version 25.0.

**Results:**

The complete 24-hour guidelines were met only by 24.4% of sample (*n* = 61). Sedentary behavior was the most met guideline (88%), followed by MVPA (56.8%) and sleep (53.2%), without significant difference according to sex. When compared to elderly participants, those participants in younger groups (18–39 years and 40–59 years) were more likely to meet MVPA guideline (PR = 2.51 [95%CI = 1.47; 4.28] and PR = 2.60 [95%CI = 1.52; 4.45], respectively), as well as the combination of MVPA and sedentary behavior (PR = 1.98 [95%CI = 1.13; 3.44] and PR = 2.17 [95%CI = 1.25; 3.79], respectively) and MVPA with the sleep guideline (PR = 2.39 [95%CI = 1.09; 5.27] only for 18–39 years group). Men were more likely to meet MVPA guideline than women (PR = 1.29 [95%CI = 1.04; 1.59]).

**Conclusion:**

Younger aged and male adults were more likely to meet the Canadian 24-hour guidelines in a small Brazilian city. However, further studies with larger and representative samples of sociodemographic stratum are still needed.

## Introduction

Physical activity is any body movement produced by skeletal muscles that requires energy expenditure [1], being important in the prevention of diseases [2]. Not meeting global physical activity recommendations is one of the major public health concerns in the 21st century [3], being considered as the fourth leading cause of death in the world, responsible for 5.3 million deaths annually [4, 5], being related to increased risk of obesity, type 2 diabetes, some types of cancer, reduced life expectancy and risks of mental health problems [6].

In addition to not meeting physical activity recommendations, elevated spent in sedentary activities has been considered as a potential health risk factor [7]. The sedentary behavior is defined as any waking behavior with an energy expenditure lower or equal than 1.5 metabolic equivalents performed in sitting or reclining posture [8]. It is important to highlight that physical activity is an essential human act resulting from urges to feel, to explore, to transform and to connect, with distinct and integrated potentialities, interplaying with social, political and situated forces [9].

The insufficient levels of physical activity and elevated sitting time have become a global public concern, affecting millions of individuals in developed and developing countries due to social, economic, and environmental changes [10], being estimated that 27.5% of the world’s adult population do not meet physical activity recommendation for health [11] and that adults spend around 8 h per day in sedentary activities [12]. A dose-response meta-analysis reported that sedentary behavior was associated with several major chronic disease outcomes, all-cause and cardiovascular mortality, independently of physical activity [13]. However, physical activity showed to modify the association of sedentary behavior with cardiovascular and cancer mortality, especially among those with higher amount of sedentary time [14]. National and international recommendations for physical activity with a focus on public health have been developed, informing the type, quantity and intensity of physical activity necessary to maintain and improve health in the general population [15]. Those subjects who did not reach the values suggested in the Physical Activity Guides were called insufficiently active [16].

The World Health Organization (WHO), in its global recommendation, advises the practice of 150 min per week of moderate to vigorous physical activity [2], which corresponds to only 2% of waking time [17], while the other 98% of time is spent on sedentary behavior, light intensity activities (value between 1.5 and 3.0 MET’s) [7], besides the sleep time. The WHO recommendation defines as moderate-intensity those physical activities with an energy expenditure between 3 and 5.9 metabolic equivalent of task (MET), and as vigorous-intensity those activities equal to or greater than 6 MET’s [7]. The benefits of practicing moderate and vigorous physical activity are well established in the literature, but increasing evidence suggests that sleep and sedentary behavior also have important health consequences [18].

Recently, a new paradigm has emerged, which employs 24-hour Movement Guidelines, combining guidelines for all-day movement behaviors: moderate and vigorous physical activity, sedentary behavior (activity that requires a value equal to or less than 1.5 equivalent metabolic task (MET) [8, 19] and sleep, Canada becomes the first country to make timing recommendations regarding these behaviors for adults [20]. The integration of the time spent in moderate-to-vigorous physical activity, sedentary behavior and sleep into a single set of Guidelines reflects the importance of considering the activities performed throughout the day for optimal health [21].

Launched in October 2020, the Canadian 24-Hour Movement Guidelines set measurable goals for surveillance, as well as providing guidance to researchers, healthcare professionals and the general public [22]. Adults are advised to limit sedentary time to 8 h or less, including no more than 3 h of recreational screen time [20]; that sleep time is 7–9 h a day; and that 150 min per week of moderate or vigorous physical activity are accumulated [23].

Given the importance of understanding the influence of 24-hour movement recommendation on the health and well-being of the population [22], the present study intends to identify the sociodemographic profile of individuals who adhere (in isolation, combined or integral) or not, to the Canadian 24-Hour Movement Guidelines, which was the first guideline to provide time-specific recommendations for physical activity, sedentary behavior and sleep, being previously used in recent studies with Latin American adults [24–26], because there is still no similar guideline specifically for this population. Furthermore, the identification of profile and associations of meeting 24-hour movement guidelines with sex, age, socioeconomic status and ethnicity is important to direct health policies to vulnerable groups in Brazil, which is a continental-sized country with wide social and health iniquity. In addition, the investigation of meeting 24-hour movement guidelines in an inner city contributes to enlarge the breadth of evidence about its sociodemographic determinants, applicability and relevance in adults from different regions of the country, besides to guide local health policies.

## Methods

The present observational study with a cross-sectional design was carried out from 2018 to 2020, and comprised a sample of adults aged 18 years or older, being carried out in the city of Santo Anastácio, located in the Southeast region of Brazil [27]. This inner city has a total population of 20,475 inhabitants, being 19,044 from urban area, with a life expectancy of 73.6 years, a literacy rate of 99% and a human development index of 0.792, being borderline to be considered as good by United Nations Development Program [28].

### Sample

The following inclusion criteria were used: (i) being 18 years old or older; (ii) have signed the Informed Consent Form. Participants were excluded according to the following criteria: (i) misuse of the accelerometer; (ii) having given up between research procedures; (iii) absence of data for any variable included [29].

Considering that the approximate population over 18 years of age in the city of Santo Anastácio is 16,000 inhabitants (IBGE), a correlation value of *r* = 0.23 was adopted to calculate the sample size [30], with power of 80% and alpha of 5%, which determined a minimum sample size of 147 individuals [31]. However, anticipating possible refusals to participate in the study and loss of subjects and data, another 30% were added to the sample, which totaled a minimum of 192 subjects to be evaluated.

The individuals who composed the sample of this research were selected in a representative random sampling process, which considered the proportionality of inhabitants according to the census sectors and the randomization of households, streets and neighborhoods according to each sector of the city. That is, for sampling, the city of Santo Anastácio was divided according to the delimitation of the census tracts; out of a total of 34 sectors, 23 are located in urban areas, and only these were included in the study. The number of people to be interviewed in each of the census sectors was calculated based on the number of people residing in these sectors, considering the proportional population of each census sector. To carry out this procedure, the census tracts were divided based on the geographic map of the city of Santo Anastácio, according to the National Institute of Geography and Statistics [32]. The detailed sampling process has been previously published [27].

The neighborhoods were registered and numbered, as well as the streets and houses. Subsequently, neighborhoods, streets, and houses were drawn randomly based on the SPSS “random” function. Residents of the selected households over 18 years of age were considered eligible to participate in the study and invited to participate; if the selected individuals refused, a new household was drawn and selected, until completing the minimum sample size required for each census sector. This sample selection design was based on the 1st National Health Survey sample design [33].

### Data collection

The data collection was carried out by post-graduate students and scientific initiation scholarship holders with previous training on all the instruments to be used in this study. At first, the evaluators went to the drawn census sectors and visit the randomly selected households. A single visit face-to-face interview was carried out through the application of an electronic survey containing 20 sociodemographic questions and the delivery of the accelerometer to the research participant with the explanations and how this instrument should be used, lasting approximately 30 min. Messages were sent via SMS or messaging App reminding the participant to wear the accelerometer. The device was collected by the researcher at participant’s household in a pre-scheduled visit.

### Objective measure of sedentary behavior and moderate and vigorous physical activity

The objective measurement was performed using the Actigraph GT3X accelerometer (ActiGraph, LLC, Pensacola, FL, USA), which is an instrument used to directly assess physical activity in several studies in the health area, being considered a reliable tool and already tested against reliable methods for the assessment of physical activity such as doubly labeled water [34]. It was recommended that the accelerometers be positioned on the right side of the waist of the evaluated subjects, under or over the clothes, and that they should remain with the equipment for at least 10 h a day on five consecutive days (three days on weekdays and two on weekends). week) [35].

The ActiLife 6 program (ActiGraph, LLC, Pensacola, FL, USA) was used to clean the data. Each data sample, determined by counts, was summarized considering a specific time interval; this particular interval, designated as epoch, has a duration of 60 s. The period of 60-second epoch was selected to register the amount of counts per minute of activity, which is the metric used to assess the weekly volume of physical activity in different intensities [36]. Consecutive hours of zero counts and days with less than 10 h of monitoring were excluded [37].

To convert the raw data from the accelerometer into a more physiological measure, classification according to activity intensity levels was used. Thus, the cut-off point recommended by Sasaki et al. [38] for adults: sedentary activity (< 1.50 MET’s) was classified as zero counts per minute; moderate physical activity was defined as counts between 2691 and 6166 (3.01–5.99 MET’s); vigorous physical activity as the values of counts comprised in values greater than 6167 (≥ 6.00 METs).

Participants who had 150 min or more of moderate and vigorous physical activity per week, as well as a number of hours per day equal to or less than 8 in sedentary behavior were considered to meet these criteria, brought by the Canadian Movement Guideline for 24 h [23].

### Sleep

The Pittsburgh Sleep Quality Index (PSQI) [39] assesses sleep quantity and quality through a standardized questionnaire, with questions that can be easily understood and answered. This instrument was validated for the Brazilian adult population [40], and component number 3 deals with sleep duration with the following question: During the last month, how many hours of sleep did you have per night? Those who responded between 7 and 9 h of sleep met the limits brought by the recommendation of the Canadian Guide to Movement [23].

### Movement guidelines combination

The three 24-hour movement guidelines (physical activity, sedentary behavior and sleep) were analyzed individually and combined. At first, it was analyzed the meeting or not each one guideline according to independent variable categories. Secondly, paired combinations were created: (i) meeting simultaneously the guideline of physical activity and sedentary behavior, but not meeting sleep; (ii) meeting simultaneously physical activity and sleep, but not meeting sedentary behavior; and (iii) meeting simultaneously sedentary behavior and sleep, but not meeting physical activity. Third, we analyzed the association of meeting all the three 24-hour movement guidelines with independent variable categories.

### Sociodemographic aspects

The sociodemographic variables of ethnicity (White, Black, Asian, Brown/Mixed-Black or other), age (in years), sex (male or female), educational attainment and socioeconomic level were obtained through questionnaires. The ethnicity was further classified as non-White (Black, Asian, Brown/Mixed-Black or other) and White. Age was classified into age-groups: younger adults (18–39 years-old), middle-aged adults (40–59 years-old) and elderly (60 years and more). The educational attainment was assessed by the question: “What is your educational level? Responses were: i. elementary school or less; ii. high school; iii. college. The socioeconomic level was assessed by Brazilian Economic Classification Criterion [41]. This instrument considers the presence and quantity of specific and comfortable consumer goods in the residence, as well as the educational level of the participant, providing a specific score which classifies the sample into descending socioeconomic classes, from the highest to the lowest (A, B1, B2, C1, C2, D and E). The socioeconomic classes were further classified into high (classes A1 and B1), medium (classes B2, C1 and C2), and low (classes D and E) as made by previous studies [27, 29].

### Statistical analysis

**Fig. 1:**
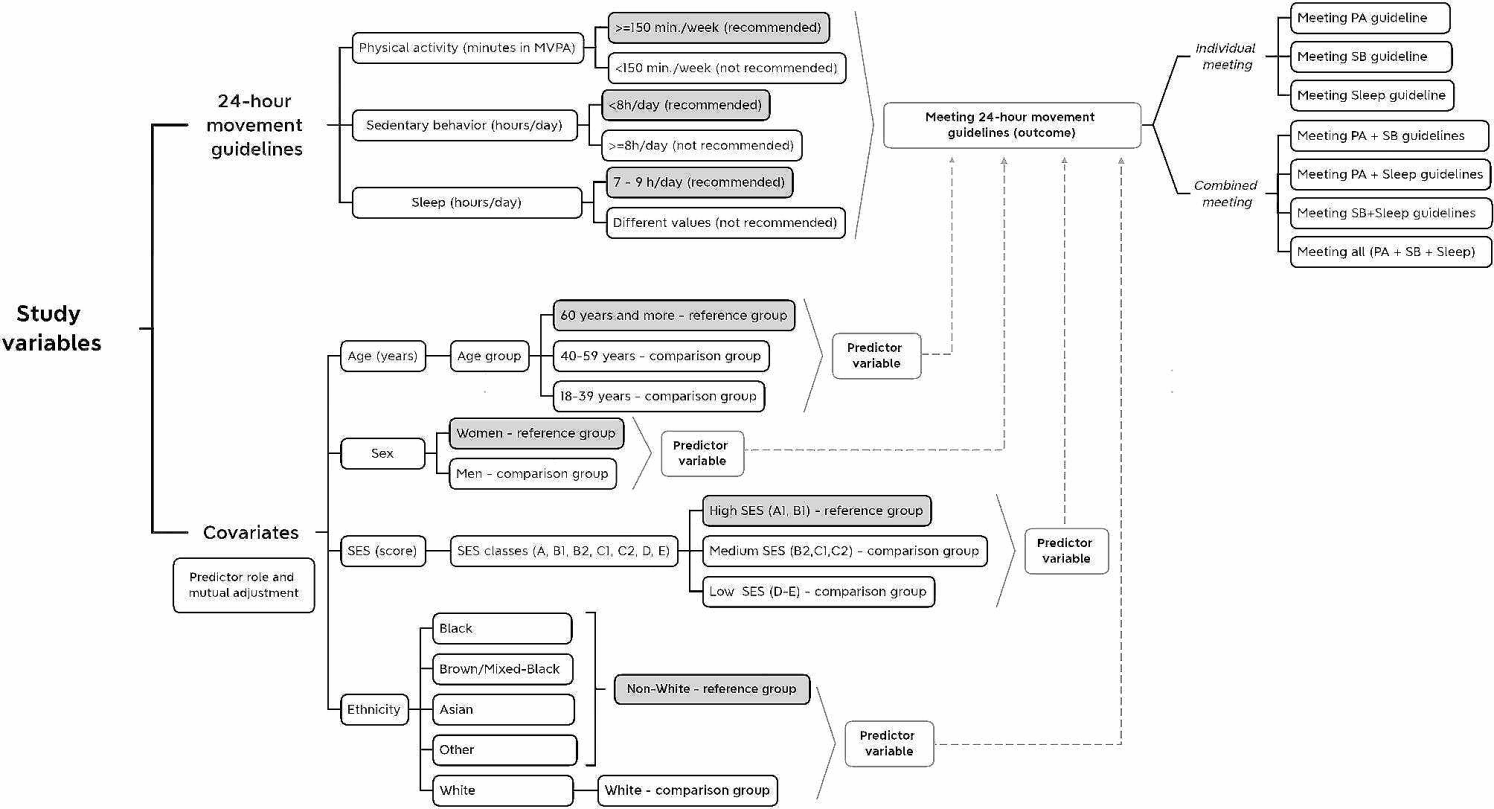
Analytical framework. legend: MVPA: Moderate-to-vigorous physical activity; SES: Socioeconomic status; PA: Physical activity; SB: Sedentary behavior

The Shapiro-Wilks test identified skewed data distribution; therefore, the variables that characterize the sample were expressed in median and interquartile range. The independent variables of this article were ethnicity, sex, age, educational attainment and socioeconomic level. Continuous variables distribution were compared into groups by Kruskal-Wallis test, whereas proportions were compared y chi-square test. Poisson regression models with a robust variance estimator were used to analyze the association between sociodemographic aspects with meeting the Canadian 24-hour Movement Guidelines, separately and combined. Statistical significance was set at *p* < 0.05 and a 95% confidence interval, with analysis performed using the SPSS statistical package, version 24.0. The analytical framework is presented in Fig. 1.

## Results

The sample consisted of 250 individuals (approximately 1.6% of the city’s adult population), being 140 females (56%) and 110 males (44%), with a median age of 41.0 (IQR = 27.0–53.0) years. There is a predominance of subjects aged between 18 and 39 years (47.6%), white (59.6%), with medium socioeconomic status (56.4%) and high school educational level (48.6%). The sample presented a median of 170 min per week of moderate-to-vigorous physical activity, six hours per day of sedentary behavior and seven hours of sleep per day. The characteristics of sample is presented in Table 1.

**Table 1:** Descriptive characteristics of the sample (*n* = 250)

Variables	Value
Sex, n (%, 95% CI)	
Men	110 (44.0, 38.0–50.2)
Women	140 (56.0, 49.8–62.0)
Age (years), median (IQR)	41.0 (27.0–53.0)
Age group, n (%, 95% CI)	
18 to 39 years	119 (47.6, 41.5–53.8)
40 to 59 years	93 (37.2, 31.4–43.3)
60 years and more	38 (15.2, 11.3–20.2)
Ethnicity, n (%, 95% CI)	
White	149 (59.6, 53.4–65.5)
Black	21 (8.4, 5.6–12.5)
Asian	6 (2.4, 1.1–5.1)
Brown	73 (29.2, 23.9–35.1)
Other	1 (0.4, 0.1–2.2)
Socioeconomic level, n (%, 95% CI)	
High	25 (10.0, 6.9–14.3)
Medium	141 (56.4, 50.2–62.4)
Low	84 (33.6, 28.0–39.7)
Educational attainment, n (%, 95% CI)	
Elementary school	57 (22.8, 18.0–28.4)
High school	121 (48.4, 42.3–54.6)
College	72 (28.8, 23.5–34.7)
MVPA (min./week), median (IQR)	170.0 (99.8–280.8)
Sedentary behavior (hours/day), median (IQR)	6.0 (4.8–7.2)
Sleep duration (hours), median (IQR)	7.0 (6.0–8.0)

IQR: Interquartile range; MVPA: Moderate to vigorous physical activity

The Table 2 presents the prevalence of meeting 24-hour movement guidelines according to sex. Regarding moderate-to-vigorous physical activity, men have a prevalence of meeting 150 min per week of 63.6%, while women were 51.4% (borderline significance p-value = 0.053). No difference was observed in meeting guidelines according to sex, neither separately nor in combination (*p*-value > 0.05).

**Table 2:** Prevalence of meeting 24-hour movement guidelines (*n* = 250)

	Overall sample (*n* = 250)			Women (*n* = 140)			Men (*n* = 110)			x²
	*n*	%	95% CI	*n*	%	95% CI	*n*	%	95% CI	*p*-value
Met PA guideline	142	56.8	50.7–62.9	72	51.4	43.2–59.6	70	63.6	54.3–72.0	0.053
Met SB guideline	220	88.0	84.0–92.0	125	89.3	83.1–93.4	95	86.4	78.7–91.6	0.480
Met Sleep guideline	133	53.2	47.0-59.3	79	56.4	48.2–64.4	54	49.1	39.9–58.3	0.248
Met PA + SB guidelines	129	51.6	45.4–57.7	68	48.6	40.4–56.8	61	55.5	46.1–64.4	0.280
Met PA + Sleep guidelines	68	27.2	22.1–33.0	33	23.6	17.3–31.3	35	31.8	23.9–41.0	0.146
Met SB + Sleep guidelines	116	46.4	40.3–52.6	69	49.3	41.1–57.5	47	42.7	33.9–52.1	0.302
Met PA + SB + Sleep guidelines	61	24.4	19.5–30.1	31	22.1	16.1–29.7	30	27.3	19.8–36.3	0.349

CI: Confidence interval; PA: Physical activity; SB: Sedentary behavior

**Fig. 2:**
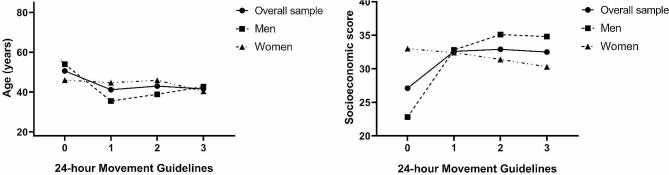
Age and socioeconomic score values according to meeting the 24-hour Guideline criteria

The Fig. 2 presents the age and socioeconomic level of the sample according to meet the 24-hour movement guidelines. There was no significant difference in age and socioeconomic score according to the clustering of guidelines met in overall sample and neither according to sex (*p* > 0.05).

The Table 3 presents the association of meeting each one of the 24-hour movement guidelines with sociodemographic variables. When compared to participants with 60 years and more, participants aged between 40 and 59 years and 18–39 years were more likely to meet the physical activity guideline (PR = 2.60 [95%CI: 1.52; 4.45]; and OR = 2.51 [95%CI = 1.47; 4.28]; respectively). According to sex, men were 29% more likely to meet physical activity guideline when compared to women (OR = 1.29 [95%CI = 1.04; 1.59]).

**Table 3:** Association of meeting individually each one of the 24-hour movement guidelines with sociodemographic aspects in adults (*n* = 250)

	Complies PA	Complies SB	Complies Sleep
	PR (CI 95%)	PR (CI 95%)	PR (CI 95%)
*Age group*			
60 years and more	Ref.	Ref.	Ref.
40 to 59 years	**2.60 (1.52; 4.45),** *p*** = 0.001**	1.14 (0.97; 1.35), *p* = 0.124	0.87 (0.65; 1.15), *p* = 0.328
18 to 39 years	**2.51 (1.47; 4.28),** *p*** = 0.001**	1.07 (0.90; 1.27), *p* = 0.464	0.87 (0.64; 1.17), *p* = 0.342
*Sex*			
Women	Ref.	Ref.	Ref.
Men	**1.29 (1.04; 1.59),** *p*** = 0.022**	0.97 (0.88; 1.06), *p* = 0.487	0.90 (0.72; 1.14), *p* = 0.384
*Socioeconomic level*			
High	Ref.	Ref.	Ref.
Medium	0.92 (0.72; 1.16), *p* = 0.456	0.99 (0.90; 1.10), *p* = 0.849	1.04 (0.81; 1.33), *p* = 0.770
Low	1.05 (0.73; 1.51), *p* = 0.794	1.04 (0.91; 1.20), *p* = 0.543	1.16 (0.80; 1.69), *p* = 0.436
*Ethnicity*			
Not white	Ref.	Ref.	Ref.
White	0.90 (0.72; 1.12), *p* = 0.328	1.01 (0.92; 1.11), *p* = 0.802	1.09 (0.86; 1.37), *p* = 0.478
*Educational attainment*			
College	Ref.	Ref.	Ref.
High school	0.98 (0.76; 1.26), *p* = 0.855	0.97 (0.88; 1.06), *p* = 0.465	1.06 (0.81; 1.40), *p* = 0.658
Elementary school	0.94 (0.69; 1.27), *p* = 0.672	0.90 (0.78; 1.03), *p* = 0.132	1.16 (0.86; 1.57), *p* = 0.342

PR: Prevalence ratio; CI: Confidence interval

The association of meeting combined 24-hour movement guidelines with sociodemographic variable is presented in Table 4. Participants in younger groups (18–39 years and 40–59 years) were more likely to meet both the recommendations for physical activity and for sedentary behavior (PR = 1.98 [95%CI = 1.13; 3.44)]; and PR = 2.17 [95%CI = 1.25; 3.79], respectively). The younger group (18–39 years) was also more likely to meet the recommendations of physical activity and sleep, when compared to the older (60 years and more) group (PR = 2.39 [95%CI = 1.09; 5.27]).

**Table 4:** Association of meeting combined 24-hour movement guidelines with sociodemographic aspects in adults (*n* = 250)

	Meet PA + SB	Meet PA + Sleep	Meet SB + Sleep	Meet PA + SB + Sleep
	PR (CI 95%)	PR (CI 95%)	PR (CI 95%)	PR (CI 95%)
*Age group*				
60 years and more	Ref.	Ref.	Ref.	Ref.
40 to 59 years	**2.17 (1.25; 3.79)** *p*** = 0.006**	2.18 (0.97; 4.91) *p* = 0.060	1.01 (0.68; 1.49) *p* = 0.978	2.32 (0.95; 5.70) *p* = 0.065
18 to 39 years	**1.98 (1.13; 3.44)** *p*** = 0.016**	**2.39 (1.09; 5.27)** *p*** = 0.031**	0.92 (0.63; 1.36) *p* = 0.686	2.36 (0.98; 5.68) *p* = 0.056
*Sex*				
Women	Ref.	Ref.	Ref.	Ref.
Men	1.14 (0.90; 1.45) *p* = 0.277	1.42 (0.96; 2.10) *p* = 0.084	0.87 (0.66; 1.14) *p* = 0.307	1.30 (0.84; 2.01) *p* = 0.248
*Socioeconomic level*				
High	Ref.	Ref.	Ref.	Ref.
Medium	0.89 (0.69; 1.16) *p* = 0.392	0.93 (0.60; 1.43) *p* = 0.733	1.06 (0.79; 1.43) *p* = 0.690	0.94 (0.58; 1.51) *p* = 0.783
Low	1.02 (0.69; 1.52) *p* = 0.912	1.17 (0.60; 2.26) *p* = 0.651	1.18 (0.76; 1.85) *p* = 0.467	1.21 (0.58; 2.52) *p* = 0.605
*Ethnicity*				
Not white	Ref.	Ref.	Ref.	Ref.
White	0.89 (0.70; 1.12) *p* = 0.318	1.03 (0.69; 1.54) *p* = 0.901	1.07 (0.81; 1.40) *p* = 0.646	0.99 (0.64; 1.56) *p* = 0.987
*Educational attainment*				
College	Ref.	Ref.	Ref.	Ref.
High school	0.97 (0.73; 1.27) *p* = 0.803	1.08 (0.67; 1.75) *p* = 0.755	1.11 (0.80; 1.53) *p* = 0.537	1.14 (0.68; 1.93) *p* = 0.640
Elementary school	0.90 (0.64; 1.28) *p* = 0.556	1.13 (0.65; 1.96) *p* = 0.669	1.10 (0.76; 1.61) *p* = 0.608	1.04 (0.55; 1.96) *p* = 0.900

PR: Prevalence ratio; CI: Confidence interval

## Discussion

The present study aimed to analyze the association of meeting the Canadian 24-hour movement guidelines with sociodemographic aspects in adults from a Brazilian inner city. As main findings, it was observed that younger age groups and males were more likely to meet physical activity guidelines than counterparts, whereas combination of physical activity + sedentary behavior and physical activity + sleep guidelines were more likely to be met by younger age groups (younger and middle-aged) than elderly.

Currently, scientific evidence on the proportion of individuals meeting the Canadian 24-Hour Movement Guidelines in its entirety is low [22]. Among inhabitants of developing countries, this information is extremely scarce [42]. The present study observed a prevalence of 24.4% of meeting the three 24-hour movement guidelines. Ferrari et al. [24] stated that their study was the first to examine prevalence and sociodemographic correlates with the 24-Hour Movement Guidelines using Latin American data, reporting that only 1.6% of adults fully complied with the recommendations. More closely to the present study findings, a study by Riquelme et al. [42] reported that 18.1% of a sample of Chilean adults fully met the 24-hour movement behavior guidelines, as well as the study of Liangruenrom et al. [43], which identified a prevalence of 21% of meeting all three 24-hour movement guidelines in a sample of Thai adults.

When the 24-hour movement guidelines were analyzed individually, it was observed that sedentary behavior was the most met guideline by the present study sample (88.0%), followed by physical activity (56.8%), being higher than previous studies. The study of Ferrari et al. [24] reported a prevalence of 48.3% of meeting physical activity guideline, whereas Riquelme et al. [42] reported a prevalence of only 22.0%. Global estimates indicate that 72.5% of adults meet the World Health Organization recommendation for physical activity [17]. Adults from developed countries have shown higher prevalence of meeting sufficient levels of physical activity than observed in present study: Europe 61%, North America 62%, Australia 60% and Canada 65% [44]. In Thailand, this prevalence reached 81.7% [43].

The physical activity guideline was more likely to be met by men than women in the present study. This finding converged with the study of Ferrari et al. [24], as well as previous studies by Carlson et al. [45], Tucker et al. [46] and Hallal et al. [47]. However, this is not fully consensual worldwide, once Marques et al. [44] stated that European women were more active than men. Different levels of physical activity according to sex can be related with the different context of physical activity in daily life, where men are more likely to be physically active at leisure than women, being mitigated when considered other contexts such as housework, occupational tasks and active commuting [48]. In addition, considering that men have biological advantages for higher physical fitness than women [49], they are more prone to have higher amount of physical activities at moderate-to-high intensity, which are those accounted for physical activity guidelines. The social determinants showed to strongly influence the physical activity at the individual level among Brazilian population [50].

Furthermore, younger participants were more likely to meet the physical activity guideline when compared to older participants in the present study. Ferrari et al. [24] reported similar results for physical activity and also for sleep guideline. The present study also observed that younger participants were more likely to meet combined guidelines of physical activity + sedentary behavior and physical activity + sleep. Several studies [45, 51, 52] previously reported that physical activity engagement decreases with age, which turns older participants less prone to meet sufficient physical activity levels. Regarding sleep, it has been reported that sleep duration tends to decrease at aging [53], which may turn older adults less prone to meet sleep recommendation. In addition, it has been observed that 67% of older adults spent more than 8.5 h per day in sedentary activities [54], also contributing to not meet the sedentary behavior guideline. Together, all these factors can help to clarify why age was the main factor associated with not meeting the 24-hour guidelines in the present study.

Regarding educational level, the study of Ferrari observed that Latin-American adults with low education were more likely to meet 24-hour guidelines than those with higher educational levels. The present study did not confirm this hypothesis, observing no association for educational attainment and for socioeconomic status either. Diverging from the findings of Ferrari et al. [24], a study with a large sample of US adults, Kindratt et al. [55] observed that participants with college degree or higher were more likely to meet 24-hour movement guidelines than those from all lower educational level groups. This lacking association among the present study sample may be due to the fact that the present study sample was recruited in a small inner city, which can be less susceptible to social inequities than larger urban centers, as capitals. Anyway, the socioeconomic level can be an important determinant of the context where physical activity is practiced, since leisure-time activities are not fully available in secure and adequate places and facilities free of charge, associated with less knowledge about health-related issues regarding physical activity [56]. Otherwise, people with lower socioeconomic levels tend to use active commuting such as walking and cycling as an alternative for more cheap transport instead of a healthier lifestyle choice [57], besides having higher physical effort at occupational activities [58]. In this sense, total moderate-to-vigorous physical activity level can be accounted from different domains of daily life, mitigating domain-specific influence of socioeconomic level.

The present study investigated the association between ethnicity and meeting 24-hour movement guidelines, observing no association. The study of Riquelme et al. [42] also considered this variable and observed that participants who reported to have indigenous ethnicity were more likely to meet 24-hour movement guidelines. The highly admixed characteristics of Brazilian sample may have compromised this association, in addition to the assessment in the present study was self-reported. A recent study of Kindratt et al. [55] reported that non-Hispanic Blacks were less likely to meet 24-hour movement guidelines when compared to those who were non-Hispanic White.

The present study has limitations, such as the lack of cross-cultural validation of 24-hour Canadian movement guidelines for Brazilian adults, once Brazil has specific recommendation for physical activity but not regarding simultaneous time-specific cutoff point of daily amount of sleep and sedentary behavior. In addition, the variables assessed by questionnaire were susceptible to response bias, such as sleep time. However, as strong points we highlight the objective measurement of sedentary behavior and physical activity, as well as the sample selection with robust criteria related to randomization and distribution, covering total geographic urban area of the city and a considerable diversity of the population, a theme that had been requested by the scientific literature due to the limited data on movement behaviors for 24 h in developing countries, such as those in Latin America. However, many other studies are still needed to establish a consensus to guide epidemiological work towards promoting a better lifestyle, identifying population profiles and more vulnerable groups. This study findings are still limited to a small city in the southeast region of Brazil, being not representative of the Brazilian population. In addition, as the sampling process of the present study did not consider the representativeness by population stratum (age, sex, educational attainment and socioeconomic status), these findings cannot be generalized to other population realities.

It is concluded that the age group was the main sociodemographic variable associated with meeting the 24-hour movement guidelines, once younger groups were more likely to meet the guideline of physical activity independently and combined with the sedentary behavior guideline, when compared to elderly participants. In addition to the age, the sex was also associated with meeting 24-hour movement guidelines, where men were more likely to meet the physical activity guideline than women. The physical activity showed to be the main guideline from the 24-hour movement behaviors associated with sociodemographic aspects in a southeastern Brazilian inner city. However, further studies with larger and representative samples are still needed to better understand the sociodemographic determinants of meeting 24-hour movement guidelines in Brazilian population.

## Data Availability

All data are available from the corresponding author upon reasonable request.
